# Dissociation between Conceptual and Perceptual Implicit Memory: Evidence from Patients with Frontal and Occipital Lobe Lesions

**DOI:** 10.3389/fnhum.2015.00722

**Published:** 2016-01-13

**Authors:** Liang Gong, JiHua Wang, XuDong Yang, Lei Feng, Xiu Li, Cui Gu, MeiHong Wang, JiaYun Hu, Huaidong Cheng

**Affiliations:** ^1^Department of Neurology, Affiliated ZhongDa Hospital, School of Medicine, Southeast University, Nanjing, China; ^2^Department of Neurology, People’s Hospital of YuXi City, The Sixth Affiliated Hospital of Kunming Medical University, Yuxi, China; ^3^The Second Affiliated Hospital of Anhui Medical University, Hefei, China

**Keywords:** implicit memory, frontal lobes, occipital lobes, explicit memory, priming, brain injury

## Abstract

The latest neuroimaging studies about implicit memory (IM) have revealed that different IM types may be processed by different parts of the brain. However, studies have rarely examined what subtypes of IM processes are affected in patients with various brain injuries. Twenty patients with frontal lobe injury, 25 patients with occipital lobe injury, and 29 healthy controls (HC) were recruited for the study. Two subtypes of IM were investigated by using structurally parallel perceptual (picture identification task) and conceptual (category exemplar generation task) IM tests in the three groups, as well as explicit memory (EM) tests. The results indicated that the priming of conceptual IM and EM tasks in patients with frontal lobe injury was poorer than that observed in HC, while perceptual IM was identical between the two groups. By contrast, the priming of perceptual IM in patients with occipital lobe injury was poorer than that in HC, whereas the priming of conceptual IM and EM was similar to that in HC. This double dissociation between perceptual and conceptual IM across the brain areas implies that occipital lobes may participate in perceptual IM, while frontal lobes may be involved in processing conceptual memory.

## Introduction

Neuropsychological studies of human memory have produced a wealth of data supporting the concept that memory is not a monolithic neural system. Instead, it consists of distinct components, which are in turn mediated by distinct and separable neural systems (Dienes and Perner, 1999; Reber, 2013). Indeed, patients coping with brain lesions, such as ischemia or traumatic brain injury, frequently demonstrate dissociated memory systems. That is, only specific forms of memory show deficits. According to the multiple memory systems theory, Tulving (1995) proposed that memory can be divided into explicit memory (EM) and implicit memory (IM) by different methods of processing information. IM refers to an individual’s past experience to influence the current task automatically but without conscious awareness of these previous experiences (Schacter, 1987). In contrast to IM, EM is characterized as being conscious and reliant on intentional retrieval. It has been determined that the performance of EM tasks, such as free recall and recognition, depends significantly upon the integrity of the medial temporal lobe (MTL) memory system, whereas performance of IM tasks, such as free-association and category exemplar generation, depends upon the integrity of other neural structures (Squire et al., 1993).

The evidence for IM derives from the studies of priming; the process means that subjects show improved performance of tasks that have been unintentionally prepared (Graf and Mandler, 1984). In IM tasks, the encoding phase is unintentional. When a recently encountered stimulus is reencountered, it is processed differently and usually more quickly. Recently encountered stimuli also show a tendency to “pop to mind” on partial cuing (Schacter, 1987). There are two general categories of implicit tests defined according to the priming type: perceptual and conceptual (Roediger and McDermott, 1993). Perceptual implicit tests draw on the physical characteristics of studied items, such as word identification and image identification tasks (Koutstaal et al., 2001; Matsukawa et al., 2005). On the other hand, conceptual implicit tests draw on the meaning or semantic associations of studied items (Blaxton, 1989), as in the case of free-association and category exemplar generation tests (Schacter and Buckner, 1998). A number of studies have demonstrated that one class of priming tasks, including word association and category exemplar generation, indexes conceptual memory processes, which are impaired in Alzheimer’s disease (AD) (Fleischman et al., 1995), while another class, including identification of words and pictures, indexes perceptual memory processes, which are localized to occipital circuits that are relatively spared in AD (Lazzara et al., 2001; Fleischman et al., 2005; Golby et al., 2005). Indeed, Fleischman et al. (2005) found that hallmark indices of AD neuropathology were associated with performance of priming tests requiring conceptual, but not perceptual, processing resources. In addition, repetition priming was also accompanied with reductions in neural activity in task-related functional magnetic resonance imaging (fMRI) study (Schacter et al., 2007). A different study found that conceptual priming was based on neural priming in the frontal cortex (Wig et al., 2005), while perceptual priming was based on neural priming in the occipital cortex (Blaxton et al., 1996; Schacter and Buckner, 1998). However, most studies gathered these results using neuroimaging methods, and the subtype of IM performance was still not clear in patients suffering from impairment of different brain areas.

The aim of the present study is to elucidate the precise neural basis of IM, using a battery of neuropsychological tests in patients suffering from injury to different brain areas. The study used structurally parallel perceptual [picture identification (PID)] and conceptual [category exemplar generation (CAG)] implicit priming tasks, as well as EM (immediate recall, delayed recall, delayed recognition) tests, in patients with frontal and occipital lobe injury, as well as cognitively normal controls. We predicted that the patients with frontal lobe lesions would exhibit conceptual IM deficits, while the patients with occipital lobe lesions would exhibit impairment in perceptual IM, relative to healthy controls (HC).

## Materials and Methods

### Participants

**Figure 1:**
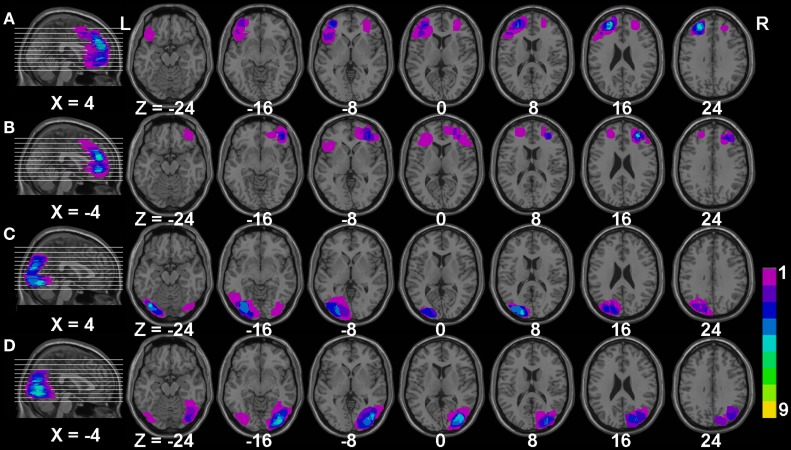
**The overlaying maps of brain injury in patient groups**. **(A,B)** The main injured regions in patients with frontal lobe injury; **(C,D)** the main injured regions in patients with occipital lobe injury. The color bar reflects the number of injured patients; purple color represents one patient while yellow color represents nine patients injured in this area. *X* and *Z* represent the Montreal Neurological Institute space (MNI) coordinates in the brain.

Brain-injured group: 20 patients with frontal lobe injury, including nine ischemic, nine hemorrhagic stroke, and two brain trauma patients, and 25 patients with injured occipital lobes, including 13 ischemic, nine hemorrhagic stroke, and three brain trauma patients, were included in this study. Brain injury was confirmed in all patients using CT/MRI scanning. The patients were hospitalized in the Department of Neurology of The People’s Hospital of YuXi City. Inclusion criteria were (1) single injury on the frontal or occipital lobe with symptoms lasting between 2 weeks and 2 months; (2) no signs of aphasia, agnosia, diplopia, or neglect; (3) no depression or psychiatric symptoms after injury, and no psychiatric history; and (4) general cognitive function rated as normal, and a mini-mental state examination (MMSE) ≥20 points. In the frontal lobe lesion group, 14 patients had hypertension and eight patients had diabetes, while 20 patients had hypertension and eight patients had diabetes in the occipital lobe lesion group. In order to clearly illustrate the region of lesion in our patients, we manually drew the outline of each patient’s brain injury and overlaid each graph using MRIcro software (http://www.mccauslandcenter.sc.edu/mricro/), as shown in Figure 1. The main impairment in the frontal lobe in our study was located in the bilateral dorsal lateral prefrontal cortex, while the main impaired occipital lobe was located in bilateral middle occipital gyrus in the present study.

Twenty-nine HC were recruited from the local community. Inclusion criteria for HC were as follows: (1) no psychiatric and neurological disorders; (2) no complaints of memory decline; (3) cooperative, with no serious physical illness; and (4) MMSE ≥26 points. Each subject underwent uniform clinical evaluation including medical history, neurological examination, and neuropsychological testing. The study was approved by the Research Ethics Committee of The People’s Hospital of Yuxi City, and written informed consents were obtained from all participants.

### Neuropsychological Background Tests and Explicit Memory

A comprehensive battery of tests, including the MMSE, the verbal fluency test (VFT), and the digit span forward (DS-F) and backward (DS-B) tests, were employed to assess the general cognitive and EM functions. The immediate recall, delayed recall, and delayed recognition trials were performed as multiple-form tests of EM. The neuropsychological test details introduced here were the same as in our previous study (Gong et al., 2010).

### Perceptual Implicit Memory Task

Perceptual priming was investigated using a picture identification test (Cermak et al., 1985). The stimuli consisted of 80 grayscale images, 40 images of common objects, and 40 corresponding mosaic images of each picture. The entity images showed animals, fruits, tools, and common objects, while the mosaic image was produced in Photoshop. The identification rate of all studied images was above 90%, while the identification rate of each mosaic image was between 50 and 70% for cognitively normal adults. During the study phase, participants were presented with a list of 20 images, and were required to rate each image on a five-point liking scale. The study list assignment was counterbalanced across the participants. The study was self-paced, but participants were told to respond as quickly as possible. During testing, which was administered 15 min after the study phase, participants were presented with 40 mosaic images half of which were studied items while the other half represented new items. The participants were asked to name each image within 10 s. Guessing was allowed, and the correct rate of identification in the unstudied and studied image group was also recorded. Priming was measured as the increase in the probability of naming a studied versus an unstudied mosaic image.

### Conceptual Implicit Memory Task

Conceptual priming was evaluated using the category exemplar generation task (Fleischman et al., 2005). During the study phase, participants were presented with 20 words belonging to two of the four categories (stationery, fruits, vegetables, and household appliances), one at a time, on individually printed cards. The word frequencies were equated between all category terms. The subjects were asked to indicate whether words represented living or non-living entities, and the study list assignment was counterbalanced across all participants. During the testing phase, administered 15 min after the study phase, the subjects were asked to generate eight examples for each of the four specified categories (two old and two new), as quickly as possible. The instructions were given without reference to the previously studied list of words. A different category was presented when the subject had generated eight examples or if the subject had failed to produce a new response in the allocated 1-min period. The experimenter recorded the words using a tape recorder. The average exemplars for the unstudied categories were used as a baseline measure of generating ability on this task. Priming was measured as the increase in the probability of producing a studied versus an unstudied category exemplar.

### Statistical Analysis

The SPSS statistical package (version 16) was used for data analysis. The chi-square test was applied in the comparison of genders in the three groups. The differences in age, education, and neuropsychological score between the three groups were assessed using one-way analysis of variance (ANOVA); *post hoc* analysis was performed between the two groups using the least significant difference (LSD) test. The two-sample *t*-test was used to identify the difference in performance between the patients with left and right lesions. Pearson’s correlation analysis was conducted to examine the association between the VFTs, explicit and IM scores. The level of significance was set as *P* < 0.05.

## Results

### Demographic and Basic Neuropsychological Tasks

As shown in Table 1, there was no significant difference between the three groups in terms of age, education, or gender (*P* > 0.05). The performance of all basic neuropsychological tests was different in the three groups (MMSE, *F* = 39.95; DS-F, *F* = 14.58; DS-B, *F* = 6.53; VFT, *F* = 26.81; all *P* < 0.01). The *post hoc* test showed that the performance of patients in the frontal lobe lesion group was significantly worse than that of the HC group on the MMSE, VFT, DS-F, and DS-B tests (DS-B, *P* < 0.05; other *P* < 0.01). There was no significant difference between the occipital lobe lesion group and the HC in all basic neuropsychological tasks (*P* > 0.05), as shown in Table 1.

**Table 1:** **Demographic and neuropsychological performance in all groups**.

Characteristic	Patients with frontal lobe lesion (*n* **=** 20)	Patients with occipital lobe lesion (*n* **=** 25)	Healthy controls (*n* **=** 29)	*F* or **χ**^2^ value	*P* value
Gender (F/M)	10/10	14/11	16/13	0.67	0.72^†^
Age	63.70 ± 12.67	60.88 ± 11.01	64.93 ± 8.48	1.01	0.37
Education	8.40 ± 2.50	8.88 ± 2.52	9.86 ± 2.91	1.94	0.15
MMSE	25.95 ± 2.44**	29.12 ± 0.93	29.41 ± 0.63	39.95	0.00
DS-F	6.55 ± 1.19**	7.44 ± 0.51	7.69 ± 0.47	14.58	0.00
DS-B	3.40 ± 0.82*	3.96 ± 0.94	4.21 ± 0.56	6.53	0.00
VFT	10.30 ± 4.11**	16.92 ± 2.22	15.66 ± 3.12	26.81	0.00
Lesion hemisphere (left/right/bilateral)	9/9/2	10/14/1	–		
Hypertension (yes/no)	14/6	20/5	–	0.60	0.43^†^
Diabetes (yes/no)	8/12	8/17	–	0.31	0.57^†^

*^†^The *P* value was obtained by the chi-square test; other *P* values were obtained by one-way ANOVA analysis. Unless otherwise indicated, the measurement data are presented as mean ± SD*. *MMSE, mini-mental state examination; DS-F, digital span forward; DS-B, digital span backward; VFT, verbal fluency test*.

*Compared with healthy controls, *P* < 0.05.

**P < 0.01.

### Performance of the Explicit Memory Task

The performance on all three EM tests was different in three groups (immediate recall, *F* = 9.19, *P* < 0.01; delayed recall, *F* = 9.24, *P* < 0.01; and delayed recognition, *F* = 3.99, *P* < 0.05). The *post hoc* test showed that the EM performance was worse in patients from the frontal lobe lesion group than in patients from the HC group. The difference was statistically significant (delayed recognition, *P* < 0.05; other *P* < 0.01). There were no significant differences among the results from all three EM tasks (*P* > 0.05) obtained for patients in the occipital lobe lesion group and the HC group, as shown in Table 2.

**Table 2:** **Explicit and implicit memory task scores of the three groups**.

Measurement	Patients with frontal lobe lesion	Patients with occipital lobe lesion	Healthy controls	*F* value	*P* value
**Explicit memory**					
Immediate recall	4.00 ± 1.59**	5.04 ± 0.98	5.48 ± 1.06	9.19	0.00
Delayed recall	6.05 ± 3.07**	8.64 ± 1.68	8.21 ± 1.63	9.24	0.00
Delayed recognition	22.85 ± 4.54*	26.00 ± 4.16	25.31 ± 3.17	3.88	0.03
**Implicit memory**					
Correct rate of unstudied mosaic images (%)	35.50 ± 6.86	37.20 ± 9.47	38.79 ± 9.88	0.79	0.45
Image identification (%)	21.50 ± 6.84	5.64 ± 8.40**	21.97 ± 5.11	46.23	0.00
All exemplars for unstudied category	7.87 ± 0.28	7.88 ± 0.26	7.90 ± 0.25	0.05	0.95
Number of unstudied category exemplars generated	0.72 ± 0.57	0.82 ± 0.67	1.05 ± 0.52	2.03	0.14
Number of studied category exemplars generated	2.00 ± 1.12**	3.52 ± 0.82	3.66 ± 0.53	27.11	0.00
Category exemplar generation (%)	15.31 ± 10.03**	34.25 ± 12.25	30.60 ± 8.89	20.14	0.00

*The *P* values were obtained by one-way ANOVA analysis. The measurement data are presented as mean ± SD*.

*Compared with healthy controls, *P* < 0.05.

**P < 0.01.

### Performance of the Implicit Memory Task

The performance of both IM tests was different in the three groups (image identification, *F* = 46.23, *P* < 0.01 and category exemplar generation, *F* = 27.11, *P* < 0.01). The *post hoc* test indicated that the performance of the category exemplar generation task in the frontal lobe lesion group was worse than that in the HC group, while the performance of the image identification task in the occipital lobe lesion group was worse than that in the HC group (*P* < 0.01). There was no significant difference between the occipital lobe lesion group and the HC group in the exemplar generation task, or the image identification task between the frontal lobe lesion and the HC group (*P* > 0.05). In addition, the correct identification rate of unstudied mosaic images was similar between three groups (*P* > 0.05), and the average generated examples of unstudied categories were similar among three groups (see Table 2).

### Neuropsychological Performance between the Left and Right Lesion Patients

In order to further identify whether the laterality lesion influence the behavior performances, we compared all neuropsychological test scores between left and right lesion patients. As illustrated in Table 3, there was no significant difference on all behavior performance between left and right lesion patient (all *P* > 0.05).

**Table 3:** **Neuropsychological performance among patients with left and right frontal and occipital lobe lesions**.

Measurement	Frontal lobe lesion		Occipital lobe lesion	
	Left (*n* = 9)	Right (*n* = 9)	Left (*n* = 10)	Right (*n* = 14)
MMSE	25.00 ± 4.89	22.33 ± 4.66	28.90 ± 0.99	29.28 ± 0.91
DS-F	7.00 ± 1.41	6.22 ± 0.83	7.40 ± 0.52	7.43 ± 0.51
DS-B	3.78 ± 0.83	3.22 ± 0.67	3.90 ± 0.99	4.07 ± 0.92
VFT	9.56 ± 3.57	11.00 ± 5.10	16.80 ± 2.53	17.07 ± 2.13
Immediate recall	4.44 ± 1.81	3.67 ± 1.50	5.10 ± 0.99	5.00 ± 1.04
Delayed recall	6.44 ± 3.21	5.89 ± 3.37	8.90 ± 2.13	8.25 ± 2.51
Delayed recognition	23.14 ± 6.05	19.74 ± 8.12	25.90 ± 4.06	8.56 ± 1.34
Image identification (%)	23.22 ± 4.29	23.44 ± 5.03	7.78 ± 9.14	4.96 ± 7.55
Category exemplar generation (%)	19.47 ± 6.34	11.73 ± 2.59	35.37 ± 9.79	33.05 ± 9.32

*MMSE, mini-mental state examination; DS-F, digital span forward; DS-B, digital span backward; VFT, verbal fluency test*.

### Correlation between the Verbal Fluency Test and the Explicit and Implicit Memory Tasks

**Figure 2:**
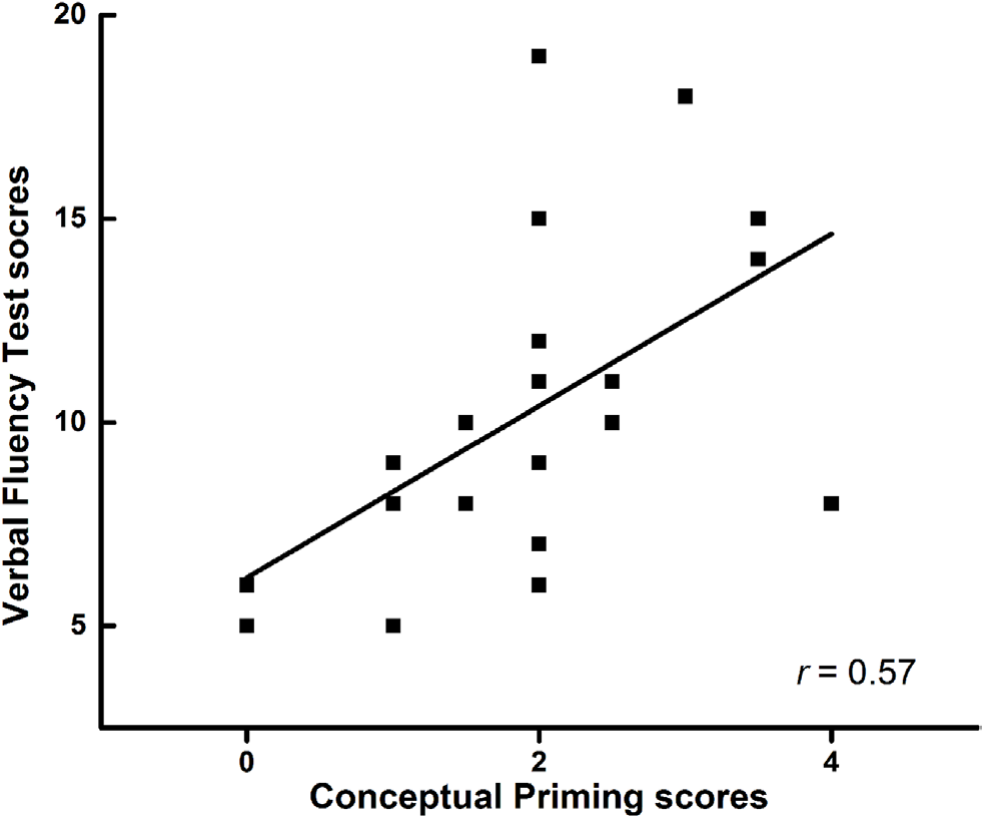
**The correlation between verbal fluency test and conceptual priming in patients with frontal lobe injury**.

The verbal fluency score was not associated with the EM score (*P* > 0.05) in all groups. The EM tasks were not associated with the IM tasks in all groups (*P* > 0.05). The verbal fluency score was not associated with perceptual IM in frontal lobe lesion patients (*r* = 0.29, *P* = 0.22). However, conceptual IM (category exemplar generation) was positive correlated with the verbal fluency score in frontal lobe lesion patients (*r* = 0.57, *P* = 0.01) (Figure 2).

## Discussion

In the current study, those patients who experienced injury to different parts of the brain were assessed for the effects of injury on IM priming, through conceptual and perceptual priming experimental paradigms, in an attempt to isolate components of IM to different parts of the brain. The results have shown that frontal and occipital lobe injuries result in a certain degree of cognitive impairment, although cognitive disorder in patients with occipital lobe injury is relatively light. The patients with frontal lobe injury exhibited a certain degree of impairment in all EM tasks, while EM was relatively preserved in patients with occipital lobe injury. It has been suggested that the frontal lobe may be involved in EM processing, which is consistent with previous findings (Fletcher and Henson, 2001). In addition, the frontal cortex may participate in episodic memory encoding (Kelley et al., 1998; Fletcher and Henson, 2001). The patients with frontal lobe injury were also impaired in the VFT and the digital span forward and backward tasks. These results indicate that the frontal lobe is involved in attention and working memory encoding as well (Smith and Jonides, 1999). The most critical result of this study is the finding that the conceptual priming task score (category exemplar generation) in patients with frontal lobe injury was lower than that in the HC group, although perceptual priming (picture identification) was similar between the two groups. In contrast, the perceptual priming task score in patients with occipital lobe injury was lower than that in HC, while performance in the conceptual priming tasks was similar to that observed in HC. In addition, there was no difference in the identification rate of unstudied mosaic images, as well as no difference in generated examples of unstudied categories among all groups. Therefore, there was a small possibility that IM impairment in patients with brain lesions contributed to poorer generation and perception ability, in comparison to HC subjects. Taken together, these data demonstrate double dissociation between conceptual and perceptual priming in patients with frontal and occipital lobe injuries, and provide strong evidence that these two priming processes are distinct from one another.

Recently, an increasing number of behavioral and neuroimaging studies has determined that the frontal lobe primarily mediates conceptual priming. For example, Wig et al. (2009) limited the activation of the left prefrontal cortex through repetitive transcranial magnetic stimulation (rTMS), thus creating a frontal lobe injury model. When analyzed with fMRI, they found that the semantic priming in the rTMS group was significantly lower than in the control group, and that the IM task-related activation of semantic priming in the left frontal area was reduced. Eskes et al. (2003) found that word fragment completion priming was impaired by left dorsolateral lesions and that category exemplar cued recall was impaired by left dorsolateral and medial lesions, while remaining priming tests were unaffected by any lesion. The results suggested that prefrontal cortex might be involved in both implicit and explicit retrieval mechanisms under certain conditions, such as those requiring lexical retrieval. The main affected area in our study was the dorsal prefrontal cortex; the present study provides further behavioral evidence for the involvement of the dorsal prefrontal cortex in conceptual priming of IM.

Many studies have shown that the performance of perceptual IM tasks is relatively spared in aging, or in patients with Parkinson’s disease (PD) and AD (Koivisto et al., 1996; McGeorge et al., 2002; Fleischman et al., 2005; Golby et al., 2005). These results imply that priming of perceptual identification might rely on the perceptual memory system, which can resist not only the impairments of EM but also widespread cognitive deterioration induced by the neurodegenerative processes in AD or PD. Few reports have found perceptual priming to be affected in patients with occipital cortex injuries. Wagner et al. (1998) reported a patient with right occipital lobe injury, in whom visual priming effects were affected but familiar recognition was well-preserved, suggesting that the right occipital lobe may contribute to perceptual priming. In the present study, the patients with lesions in the occipital cortex demonstrated impaired perceptual priming (picture identification), but in the scores in EM tasks, the patients with occipital injury performed as well as HC. These results demonstrated that the implicit and EM processes were dissociated in patients with occipital lobe injury.

The VFT score was positively correlated with conceptual priming in patients with frontal lobe lesion, but was not correlated with perceptual priming or EM tasks. We wonder if the results might be considered in the context of componential view of memory – the Component Process Framework (Cabeza and Moscovitch, 2013). This can be viewed in the context of processing components, which are associated with different neural regions and recruited in a flexible and often task-specific manner to achieve a particular processing goal. In these two tasks, the frontal executive component requiring strategy selection and maybe response inhibition can be seen as activated or recruited for both conceptual tasks.

The findings in the present study add to a growing body of neuropsychological evidence and demonstrate that the brain utilizes multiple memory systems. Two major findings emerged from this study. First, in the occipital injury group, EM was spared but perceptual priming was impaired. In contrast, in the frontal injury group, EM was impaired but perceptual priming was preserved. The double dissociations between perceptual implicit and EM provide crucial evidence that implicit and EM processes are not subserved by a single neural system, but that at least one form of IM is supported by a neural system separate and independent of the system that supports EM. Second, patients with occipital and frontal lobe injuries demonstrated double dissociation between the perceptual and conceptual priming tasks. These results were also in accordance with previous neuroimaging studies (Badgaiyan et al., 2001; Schacter et al., 2007; Wig et al., 2009). The double dissociation between perceptual and conceptual priming effects provides strong evidence for the existence of two priming processes that depend upon separate neural systems. The frontal lobes may be processing conceptual priming, while the occipital lobes may be participating in perceptual priming.

There are several limitations to this study. First, this exploratory research was conducted as a cross-sectional study with a relatively small sample size. The lesion regions exhibited heterogeneity in two patient groups, but further studies on a larger sample of patients is needed to delineate more clearly the brain regions involved in different types of IM processes. Second, the majority of patients in our study suffered from underlying disorders such as hypertension and diabetes, which also impaired cognition, especially EM (Barbagallo and Dominguez, 2014; Haring et al., 2015). In our study, the proportion of underlying disorders was similar in patients with frontal and occipital lobe lesions, so these disorders probably did not significantly influence our results. Third, we did not include additional IM tasks, in order to limit the interaction effect between EM tasks and priming tasks. In correlation analysis, due to a small sample of this study, we did not use the multiple comparison correction. Finally, we did not use neuroimaging technology in our study, which would provide more convincing support for our thesis.

## Conclusion

In conclusion, the findings reported here point to double dissociation between perceptual and conceptual IM, namely occipital lobes are likely involved in perceptual IM, while frontal lobes are likely involved in conceptual IM.

## Conflict of Interest Statement

The authors declare that the research was conducted in the absence of any commercial or financial relationships that could be construed as a potential conflict of interest.
